# A case report of gastric volvulus, a rare cause of acute abdomen

**DOI:** 10.1016/j.radcr.2021.04.059

**Published:** 2021-05-26

**Authors:** Abdul Qadir Qader, Hamzaini Abdul Hamid

**Affiliations:** a Radiology Department of Medical Faculty of Herat University, Afghanistan; b Radiology Department of Hospital Universiti Kebangsaan Malaysia, UKM. Kuala Lumpur, Malaysia

**Keywords:** Gastric volvulus, Organoaxial, Mesenteroaxial, CECT in gastric volvulus

## Abstract

Gastric volvulus is an uncommon disorder with an unknown incidence, unless it stays in the back of the diagnostician's mind, diagnosis of gastric volvulus, which can have significant morbidity and mortality associated with it, can be easily missed and can present either in the acute or chronic setting with variable symptoms. When it occurs in the acute scenario, patients present with severe epigastric pain and retching without vomiting. Together with inability to pass nasogastric tube, they constitute Borchardt's triad. The presence of a hiatal hernia with persistent vomiting despite initial antiemetic treatment should trigger one to think of gastric volvulus, despite the patient appearing very stable.

We report a case which presented in our hospital with abdominal pain and vomiting. As Oesophagogastroduodenoscopy shows hiatal hernia and peptic ulcer.

Primary gastric volvulus occurs in the absence of any defect in the diaphragm or adjacent organ pathology and may be caused by weakening of gastric supports.

As conclusion; Gastric volvulus is a surgical case, requiring early diagnosis and aggressive management, as a delay results into complications like gangrene and perforation which substantially increase the morbidity and mortality in these patients, and contrast enhanced computed tomography (CECT) is the best modality for diagnosis of gastric volvulus.

## Introduction

Gastric volvulus is an abnormal rotation of the stomach by more than 180°. Gastric volvulus, organo-axial or mesenterico-axial, may present either as surgical emergency or as chronic abdominal symptoms. It is a rare clinical entity that is difficult to diagnose and can be fatal in the acute scenario. Borchardt's triad of severe epigastric pain, retching and inability to pass a nasogastric tube is present in 70% cases and is believed to be diagnostic for acute gastric volvulus [1]. Complications include gastric ischemia, gangrene, perforation, pancreatic necrosis [2], omental avulsion [3] and even splenic rupture [4] in few cases. The rarity of the disease accounts for the associated high mortality (3050%) and hence requires high index of clinical suspicion [5]. A prompt and correct diagnosis followed by immediate surgery remains the key factor in reducing the morbidity and mortality. It is usually suspected on plain film radiograph of either chest or abdomen and confirmed by barium studies and CT scans. We report a case of and briefly discuss the clinical and radiological features of gastric volvulus.

## Case report

An 81 years old Malay woman diagnosed with history of hiatus hernia as Oesophagogastroduodenoscopy showed type 4 grade 4 hiatus hernia and peptic ulcer and specimen of body of stomach showed gastric body mucosa composed of regularly spaced tubular glands and vascular congestion within the lamina propria with no evidence of increased inflammatory cells or intestinal metaplasia seen. No evidence of ulcer, dysplasia or malignancy and diagnosis of body of stomach biopsy was within normal limits.

Presented with acute epigastric pain without radiation, which was associated with nausea and emesis which was non-bilious and non-bloody. On review of systems, she denied any diaphoresis, shortness of breath, constipation, diarrhoea, previous similar episodes, and use of non-steroidal anti-inflammatory drugs and alcohol.

On physical examination, she was found to be afebrile, with stable vital signs and abdominal examination revealed epigastric tenderness and severe retching and inability to pass a nasogastric tube with normal active bowel sounds.

## Imaging findings and diagnosis

**Fig. 1:**
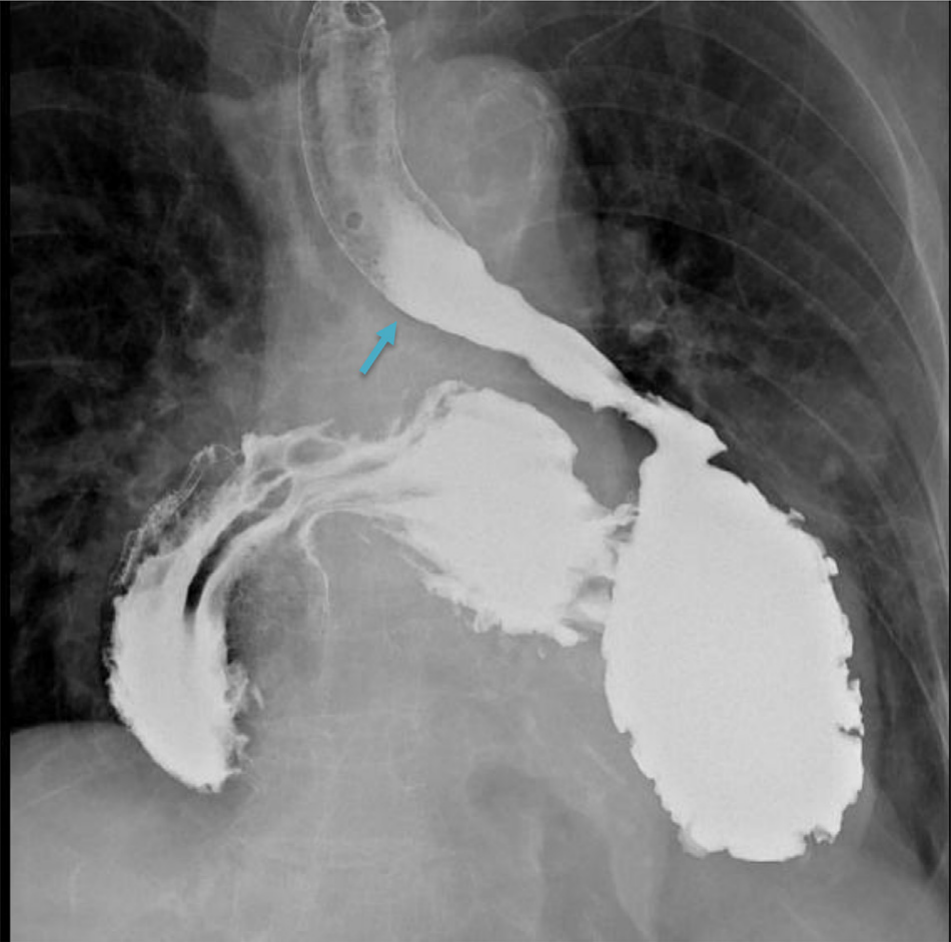
Smooth flow of contrast from the oesophagus (arrow). No dilatation of the oesophagus.

**Fig. 2:**
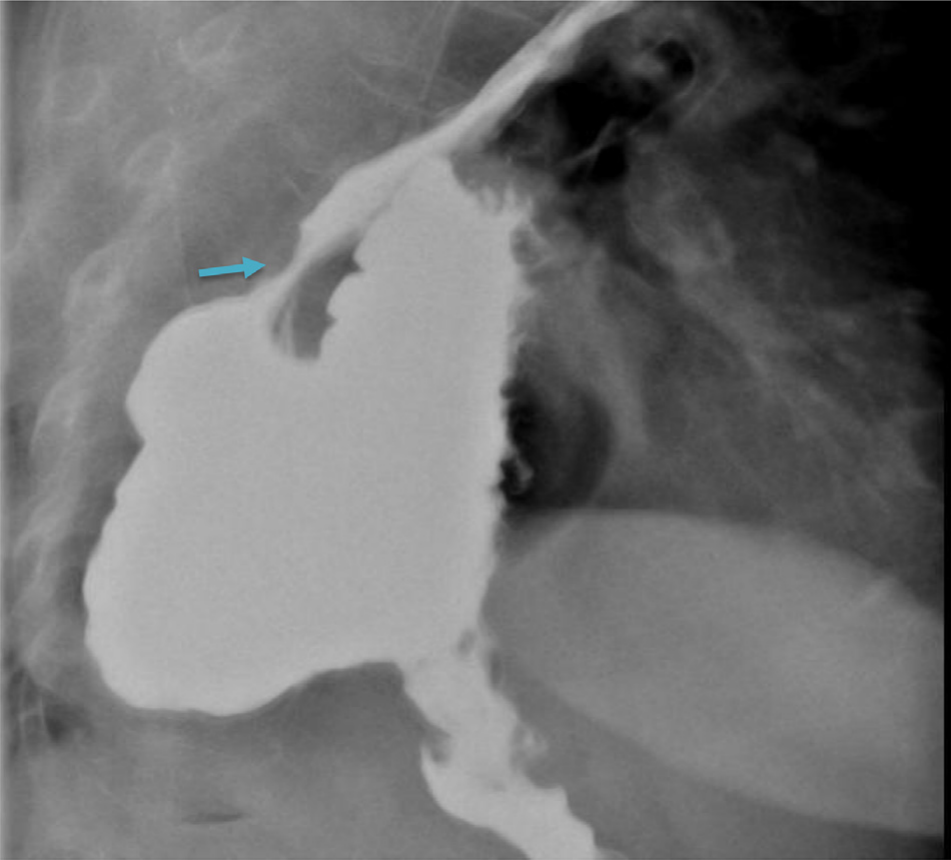
The stomach is entirely intrathoracic. The gastro-oesophageal junction is on the left side within the mediastinum (arrow).

**Fig. 3:**
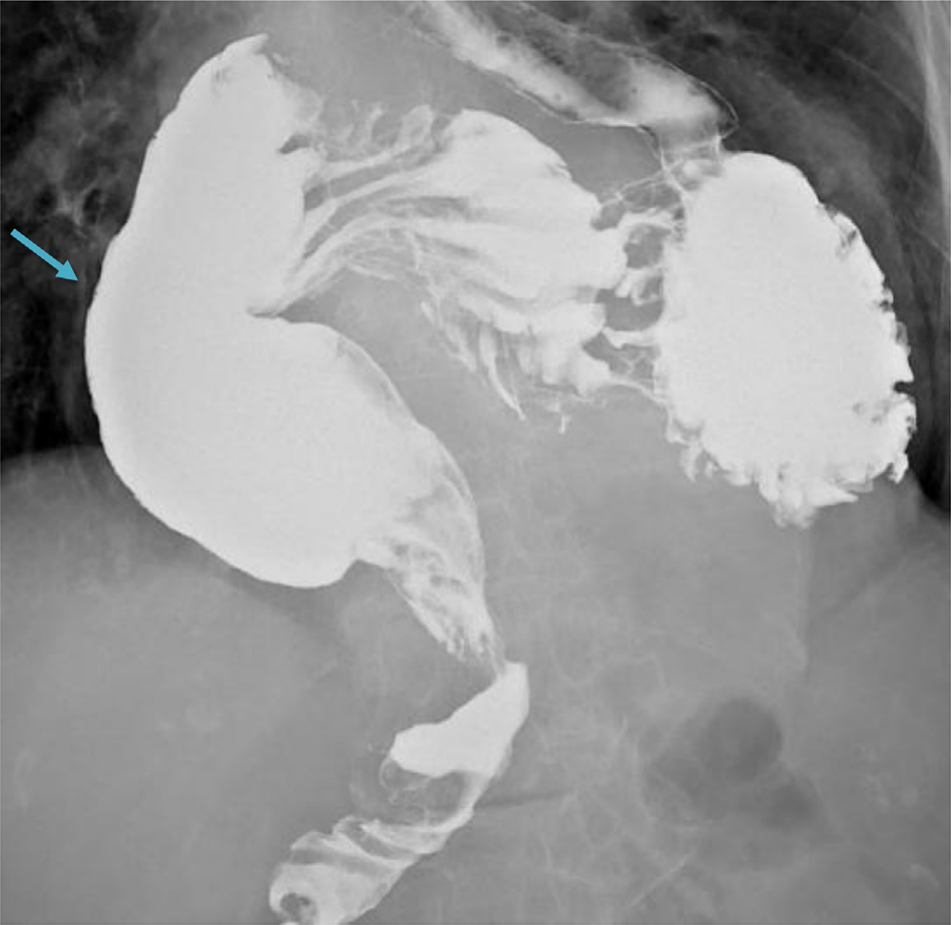
The gastric fundus is located posteriorly, while the greater curvature and the lesser curvature are reversed (arrow showing greater curvature) on the right side of the mediastinum. The pylorus lies at the expected location of gastro-oesophageal junction.

An upper gastrointestinal barium study showed smooth flow of contrast into small bowel from the oesophagus. (Fig. 1) and no dilatation of the oesophagus. The stomach is entirely intrathoracic (Fig. 2). The gastro-oesophageal junction is on the left side within the mediastinum.  The gastric fundus is located posteriorly, while the greater curvature and the lesser curvature are reversed on the right side of the mediastinum. The pylorus lies at the expected location of gastro-oesophageal junction (Fig 3).

**Fig. 4:**
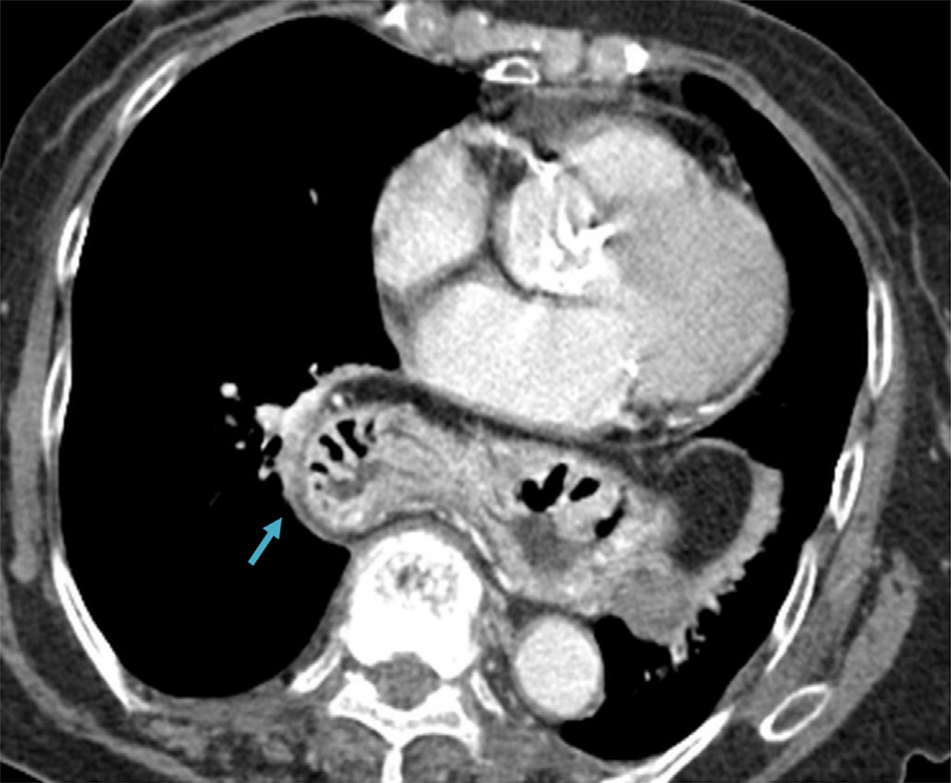
A large hiatal hernia noted, posterior to the heart. The stomach is seen herniated within the herniated sac. (arrow).

**Fig. 5:**
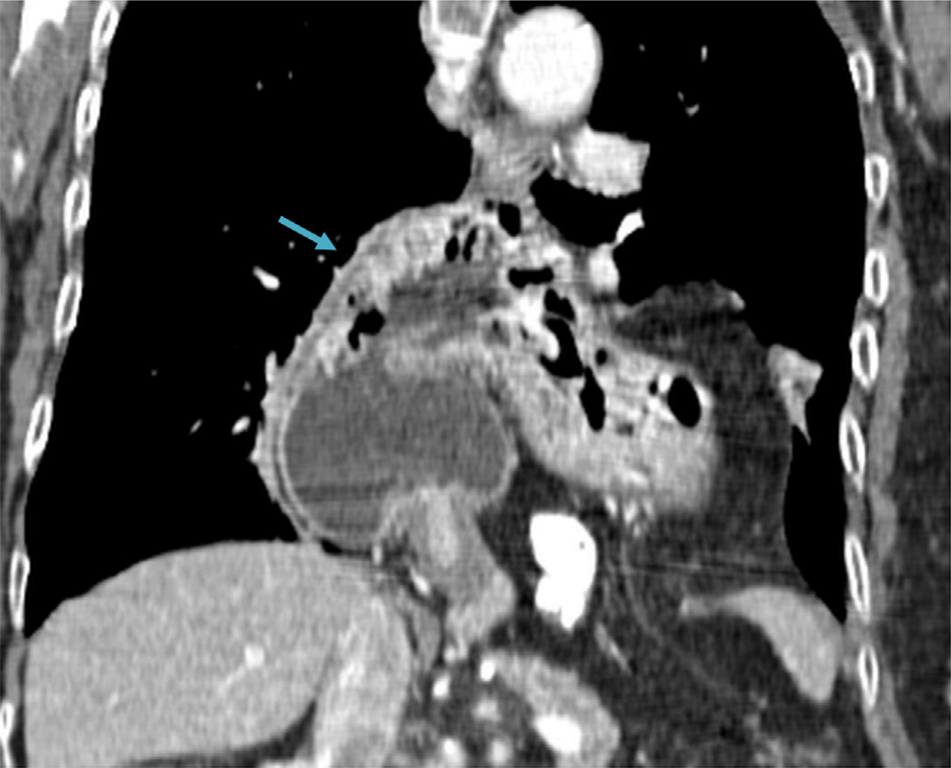
The greater curvature is superiorly located than the lesser curvature (arrow). Gastroduodenal and gastroesophageal junction are not in opposite upside-down location.

**Fig. 6:**
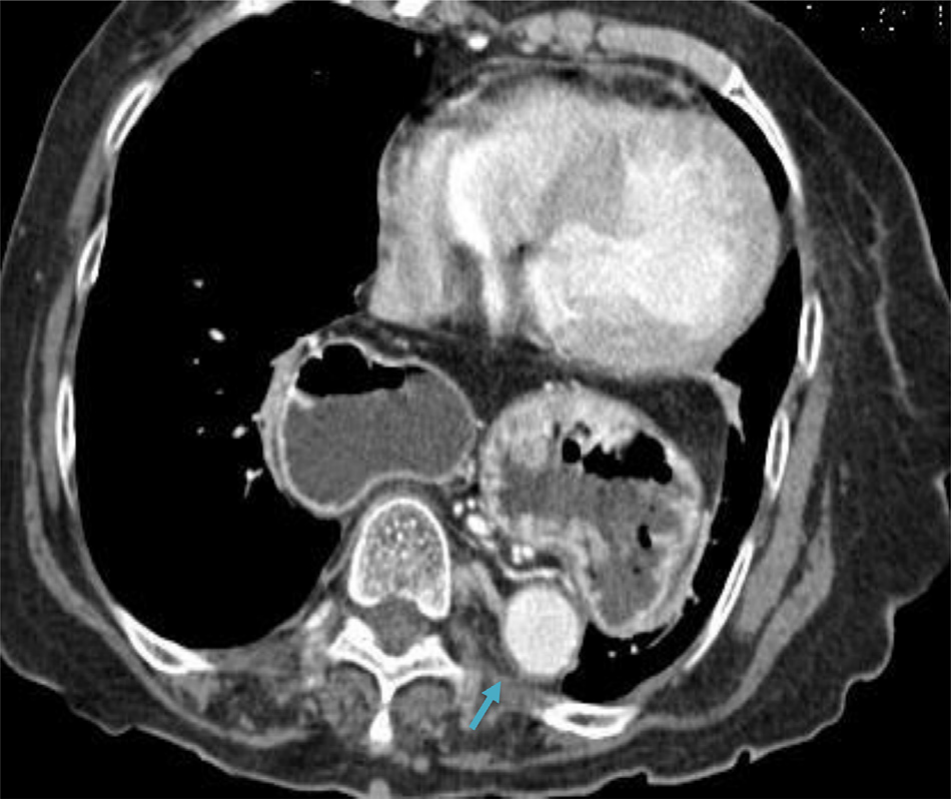
The thoracic aorta is pushed posterolaterally (arrow).

Contrast Enhanced Computed Tomography exhibited a large hiatal hernia noted posterior to the heart (Fig. 4). The stomach is seen herniated within the herniated sac and greater curvature is superiorly located than the lesser curvature (Fig. 5). Gastroduodenal and gastroesophageal junction are not in opposite upside-down location. The thoracic aorta is pushed posterolaterally (Fig 6).

## Discussion

Volvulus is described as more than 180° rotation of a hollow viscus around its mesentery, resulting in obstruction, impairment of vascularity and eventually ischemia. Most common organ to undergo volvulus in adults is sigmoid colon, followed by caecum to a lesser extent. Gastric volvulus is very uncommon, usually presenting in the 5th decade [6]. It can present either in the acute or chronic form. Acute gastric volvulus may lead to gangrene in 5%-28% of the patients [7]. There is no sex or racial predilection [8]. The gastric volvulus was first described by Berti et al. in 1866. First successful operation of gastric volvulus was done by Berg in 1897. Diaphragmatic injury may manifest immediately with herniation of abdominal contents in to the chest or it may be delayed because of initial plugging mechanism of omentum, which may not allow the diaphragmatic rent to heal and herniation may occur after several months or years [1]. Gastric volvulus can be classified on the basis of the underlying etiology as primary or secondary.

In approximately 70% cases, it occurs secondary to anatomical or functional disorders of the stomach or of the adjacent structures like spleen and diaphragm [5]. The most common association in adults is with a paraesophageal hernia. Other causes include trauma, eventration of diaphragm [9] and phrenic nerve palsy [10]. Primary gastric volvulus occurs due to malignancy, adhesions or failure of the gastric supports namely gastrocolic, gastrosplenic, gastrophrenic and gastrohepatic ligaments.

In our case, the gastric volvulus was of primary type without any associated diaphragmatic defect or paraesophageal hernia. Based on the axis of rotation, it can be organoaxial or mesenteroaxial or combined Table 1. Organoaxial variety is the commonest (60% cases) and is characterized by rotation of the stomach about an axis passing through the gastroesophageal junction and pylorus, with greater curvature lying superior to the lesser curvature [11]. Mesenteroaxial type is less common in which stomach rotates about an axis passing perpendicular to the longitudinal axis of stomach. Stomach lies in the vertical plane with antrum and pylorus lying anterior and superior to the gastroesophageal junction. The rarest variety is the combined form. In our case, the gastric volvulus was of organoaxial type.

**Table 1:** Differences of Organoaxial and Mesenteroaxial volvulus*.

Organoaxial	Mesenteroaxial
Twist occurs along a line connecting the cardia and the pylorus along the luminal (long) axis of the stomach. Most common type and usually associated with diaphragmatic and vascular compromise	Twist occurs around a plane perpendicular to the luminal (long) axis of the stomach from lesser to greater curvature. Chronic symptoms more common Diaphragmatic defects less common

Classically, Borchardt's triad of vomiting, epigastric pain and an inability to pass an NGT should trigger one to think of gastric volvulus as the primary diagnosis. Borchardt's triad has been reported to occur in 70% of cases. However, a retrospective study on the common presentations of chronic gastric volvulus over a 5-year period has shown that dysphagia, epigastric pain and chest pain occur 29% of the time individually. Patients with acute gastric volvulus typically present with Borchardt's triad of pain in epigastrium or lower chest associated with severe retching and inability to pass a nasogastric tube. Similar presentation was noted in our case. Acute gastric volvuli carry a mortality rate of 42%-56%, secondary to gastric ischaemia, perforation or necrosis.

The chronic form of the disease presents with dysphagia, dyspepsia and intermittent pain after intake of meals. The symptoms may resemble that of peptic ulcer disease, gastritis, cholecystitis or even angina pectoris [12]. It may reduce spontaneously leading to delay in diagnosis and treatment.

A clinical diagnosis is usually difficult as the disease is very uncommon. Chest radiograph may show a retrocardiac air filled mass suggestive of an intrathoracic stomach herniating through the diaphragm, which was present in our case. Abdominal radiographs may show a single large gas shadow with paucity of distal bowel gases, consistent with a distended fluid filled stomach. In chronic cases especially associated with paraesophageal hernia, barium study is the gold standard [6]. Computed tomography of abdomen can confirm the diagnosis and also identify the transition point [13]. According to Singham et al., CT abdomen should be the first line of investigation [14]. Management of gastric volvulus depends upon its presentation, the cause and the general condition of the patient. Acute presentation requires immediate surgical intervention after correction of fluid and electrolyte imbalances. Chronic forms may be managed conservatively or repaired in a planned way with satisfactory outcomes.

In the emergency laparotomy, if the stomach is not gangrenous, reduction of the volvulus with anterior gastropexy is the most commonly performed procedure. The greater curvature of the stomach is fixed to the undersurface of the anterior abdominal wall. Partial or total gastrectomy may be required in cases of gangrene or perforation of stomach [5]. To the best of our knowledge, very few cases of gastric volvulus requiring total gastrectomy have been reported in the literature [15], [16], [17]. Several other procedures have been described in literature such as diaphragmatic hernia repair, gastropexy with division of gastrocolic ligament (Tanner's operation), fundoantral gastrogastrostomy (Opolzer's operation), repair of diaphragmatic eventration [8]. Nissen's fundoplication is done in case of hiatal hernia [18]. Meena et al. reported a similar case of mesenteroaxial volvulus with gangrene of stomach sparing the antrum. They performed an esophagogastric anastomosis with pyloric dilatation, thus preventing bile reflux and preserving some reservoir function of the stomach [19]. Jethwani et al. reported a case of strangulated gastric volvulus with diaphragmatic eventration in a young girl. Patient underwent partial gastrectomy with anterior gastropexy and repair of eventration [20].

Conservative management is recommended in chronic form of the disease especially in the elderly age group. Conservative approach involves endoscopic reduction or percutaneous endoscopic gastrostomy. However, there is a considerable risk of gastric perforation and hence patients have to be selected carefully. Minimally invasive surgical approaches include laparoscopic fundoplication and repair of hiatal and paraesophageal hernia. Laparoscopic repair is associated with lesser complications, shorter hospitalization and is particularly indicated in patients with chronic volvulus [21.

In summary, unless it stays in the back of the diagnostician's mind, gastric volvulus can be an easily missed diagnosis, which is associated with significant morbidity and mortality. As mentioned above, patients do not always exhibit unstable vital signs and distressed appearance. The presence of a hiatal hernia with persistent vomiting despite initial antiemetic treatment should trigger one to think of gastric volvulus. With the advent of CT and laparoscopic surgery, the gold standards for diagnosing and treating this disease are ever evolving.

## Conclusion

Gastric volvulus is an uncommon, and often unrecognised, surgical emergency that should be considered in patients who present to the hospital with severe epigastric pain and evidence of gastric outlet obstruction. If the diagnosis is in doubt (and this is often the case), imaging studies are important in making the diagnosis. Emergency laparotomy is needed to prevent serious complications like gangrene and perforation. This case report highlights the usefulness of upper GI barium study an abdominal CT in assisting in diagnosing this life-threatening condition. The CT allows for multi-planar demonstration of abnormal torsion of the stomach and also provides valuable insights into possible aetiologies, and predisposing factors. As CT is readily available nowadays, and should be considered the diagnostic tool of choice in any suspected gastric volvulus.

*What happened to our case following CT scan diagnosis, surgery team performed surgical repairing including stomach detorsion and gastropexy and stomach strangulation or necrosis were prevented due to early diagnosis by CT scan.*

## Patient Consent Statement

Concerned patient gave consent to this case study.
